# Biomechanics of the parasite–host interaction of the European mistletoe

**DOI:** 10.1093/jxb/erab518

**Published:** 2021-11-26

**Authors:** Max D Mylo, Mara Hofmann, Frank Balle, Samuel Beisel, Thomas Speck, Olga Speck

**Affiliations:** 1 Plant Biomechanics Group @ Botanic Garden Freiburg, University of Freiburg, Freiburg, Germany; 2 Cluster of Excellence livMatS @ FIT—Freiburg Center for Interactive Materials and Bioinspired Technologies, University of Freiburg, Freiburg, Germany; 3 Department of Sustainable Systems Engineering—INATECH, University of Freiburg, Freiburg, Germany; 4 McGill University, Canada

**Keywords:** Digital image correlation, fracture analysis, fracture mechanics, haustorial system, mistletoe, parasite–host interaction, surface roughness, tensile strength, *Viscum album*

## Abstract

The European mistletoe (*Viscum album*) is an epiphytic hemiparasite that attaches to its host by an endophytic system. Two aspects are essential for its survival: the structural integrity of the host–parasite interface must be maintained during host growth and the functional integrity of the interface must be maintained during ontogeny and under mechanical stress. We investigated the mechanical properties of the mistletoe–host interaction. Intact and sliced mistletoe–host samples, with host wood as reference, were subjected to tensile tests up to failure. We quantified the rough fractured surface by digital microscopy and analysed local surface strains by digital image correlation. Tensile strength and deformation energy were independent of mistletoe age but exhibited markedly lower values than host wood samples. Cracks initiated at sites with a major strain of about 30%, especially along the mistletoe–host interface. The risk of sudden failure was counteracted by various sinkers and a lignification gradient that smooths the differences in the mechanical properties between the two species. Our results improve the understanding of the key mechanical characteristics of the host–mistletoe interface and show that the mechanical connection between the mistletoe and its host is age-independent. Thus, functional and structural integrity is ensured over the lifetime of the mistletoe.

## Teaser

Tensile tests and analysis of fracture surface roughness revealed that the mechanical bonding strength of mistletoe–host interfaces is age-independent. Cracks initiate along the interface at local strains of about 30%.

## Introduction

The European mistletoe (*Viscum album*) is a dioecious epiphytic evergreen hemiparasite that is attached to a branch of the host tree by an endophytic system. Whereas the exophytic leaves and branches of the mistletoe are photosynthetically active, it obtains water and mineral salts from its host tree (Heide-Jørgensen, 2008). The survival of individual mistletoes depends on the maintenance of the structural integrity of the parasite–host interface and of the functional integrity of water exchange throughout their life cycle of more than 20 years (Heide-Jørgensen, 2008) and their adaptation to the continuous growth processes of the host.

### Mistletoe occurrence

Parasitic plants are considered not only as pests that reduce host growth and fruiting, increase defoliation, and damage host wood (Piirto *et al.*, 1974; Lech *et al.*, 2020), but also as keystone resources having a large effect on the ecological community, especially by providing food for birds (Watson, 2001; Zuber, 2004; Heide-Jørgensen, 2008; Watson and Herring, 2012). Because of their epiphytic growth form, mistletoes are often omitted from descriptions of vegetation types, in contrast to their hosts, which are known as typical companion species (Zuber, 2004). *Viscum album* is widely known as a hemiparasitic mistletoe, a keystone resource, a pharmaceutical plant, and a symbol in mythology. Its geographical distribution is limited by an average temperature above 15 °C in the warmest month and an average above –8 °C in the coldest month (Skre, 1979). The subspecies *album* grows on a range of deciduous trees in many plant communities. Host trees include members of the genera *Acer*, *Populus*, *Salix*, *Tilia*, and other deciduous trees. The distribution of *V. album* increases with the presence of suitable trees as hosts, numerous birds as seed dispersers, and humans as cultivators of orchards of apple trees serving as hosts. In contrast, its abundance decreases as a consequence of being collected for Christmas decoration (Zuber, 2004) and for mistletoe therapy or by controlled decimation in host monocultures in which mistletoes can become a pest because of large population sizes with high population densities (Weber, 1993).

### Morphology and anatomy of host–mistletoe interface

The single-seeded berries of *Viscum album* are an important food source for birds in winter. Some birds (e.g. mistle thrush, fieldfare, or waxwing) ingest the berries and disperse the seed after its passage through their digestive system or, as in the case of the blackcap (*Sylvia atricapilla*), eat only the outer part of the berry and then leave the seed on the branch of the host tree (or on the mistletoe itself) (Zuber, 2004). Because of the sticky fruit pulp full of glutinous viscin, the seed is firmly glued to its host branch (Horbelt *et al.*, 2019). Over the next several months, the mistletoe develops a so-called haustorium. This parasite-specific organ establishes the connection to the host, enabling the exchange of water, mineral salts, and genetic information (Yoshida *et al.*, 2016; Teixeira-Costa, 2021). Heide-Jørgensen (2008) divides the continuous process of haustorial establishment into four stages. First, haustorial initiation starts in spring after germination with the development of a haustorium directly from the radicle. Second, the adhesive stage is characterized by the development of a holdfast that ensures that the parasite and the host are not pushed away from each other and that counteracts the mechanical aspects of penetration. The holdfast consists of lipids that are secreted by epidermal glandular cells and, after drying and hardening, effectively glue the seedlings onto the branch. Third, in the intrusive phase, a meristem develops and produces the intrusive organ, which penetrates the living tissues of the host and makes contact with the host xylem. When the intrusive organ reaches the host cambium, the establishment of the haustorial meristem is induced and active penetration stops. Instead, the haustorial meristem produces tissue simultaneously with the host cambium, forming annual rings and wedge-shaped sinkers. In addition to the sinker, several cortical strands develop that run through the host branch. Fourth, a xylem bridge differentiates during the conductive phase (Heide-Jørgensen, 2008). Hosts react to the infestation with hypertrophy of their branches at the infection site (Smith and Gledhill, 1983), likely due to manipulation of host cell differentiation by the mistletoe (Hu *et al.*, 2017). In the following years, growth of the mistletoe exophyte accompanies the spread of the endophyte within the host branch. After about 5–6 years, the development of buds and flowers allows for the determination of sex (Nierhaus-Wunderwald and Lawrenz, 1997). A recent study of *Viscum album* ssp*. album* has shown a female bias of about 76% for a population collected from a single host tree (*Aesculus flava*). However, the morphology of the endophyte exhibits little dependence on sex, but rather on the age of the mistletoe. Young plants have numerous equivalent sinkers, whereas at older stages, the sinkers merge to form a coherent wedge-shaped structure (Mylo *et al.*, 2021). The main fertile exophyte develops from the terminal bud of the seedling. Furthermore, additional shoots can grow out of cortical strands in later stages (Heide-Jørgensen, 2008).

### Mechanics of host–mistletoe interface

After many years, richly branched mistletoes can form spherical bushes of more than 2 m in diameter (Heide-Jørgensen, 2008). Thus, the mistletoe–host interface is subjected to mechanical loads from the weight of the mistletoe and from additional loads caused by wind, snow, or the weight of birds sitting and feeding on its fruits. Although the mechanical properties of the mistletoe–host attachment are of great interest, the mechanical properties of mistletoes themselves or of the mistletoe–host interface have not yet been investigated, to the best of our knowledge. Some studies refer to a change in the mechanical quality of the infected host wood, especially with regard to the subsequent use of the wood as timber. Piirto *et al.* (1974) have found a pronounced effect of parasitic dwarf mistletoe (*Arceuthobium americanum)* on the anatomical, chemical, and mechanical wood properties of the lodgepole pine (*Pinus contorta)*. Compared with wood of non-infected control trees, the mechanical properties in both infected and non-infected wood of parasitized trees deteriorate.

### Aim of the study

We have investigated the mechanical properties of the mistletoe–host interface and of the host wood under monotonic tensile loading up to final failure. For our calculations, we have taken into account the measured roughness of the fractured surface. The chosen experimental set-up allows us to make statements concerning the mistletoe–host interaction with regard to (i) its various mechanical properties (e.g. tensile strength, fractured area and axial rigidity), (ii) its dependency on the age and sex of the mistletoe, and (iii) the distribution of local surface strains under tensile load.

## Materials and methods

### Plant material

**Fig. 1.**
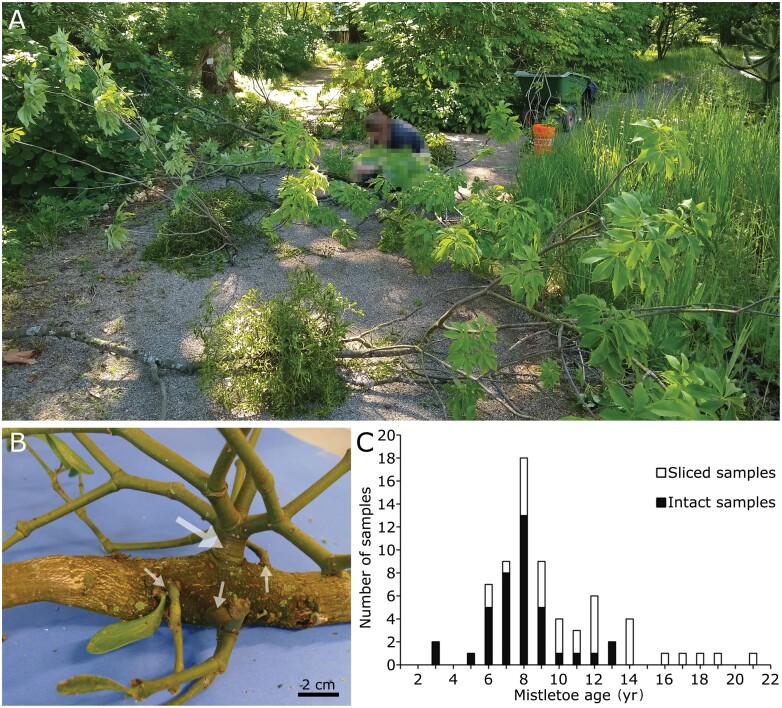
Samples of the European mistletoe (*Viscum album*) growing on *Aesculus flava* as a host tree in the Botanic Garden Freiburg (Germany). (A) Harvesting and processing of the mistletoe samples at the host branches for near-term testing. (The person involved has been blurred.) (B) Example of a mistletoe attachment to the host branch. A thickening (hypertrophy) of the host is visible in the region of the attachment site. The large arrow indicates the main basal branch of the mistletoe, which was clamped for the mechanical tests. The smaller arrows indicate shoots or younger mistletoes whose exophytes, if present, were removed prior to testing. (C) Age distribution of all evaluated mistletoe samples divided among the intact (filled bars) and the sliced (empty bars) samples.

Samples were taken from a tree of the species *Aesculus flava* SOL. (hereafter *A. flava*) growing in the outdoor area of the Botanic Garden of the University of Freiburg (Germany) (Fig. 1A), parasitized by mistletoe plants classified as *Viscum album* ssp. a*lbum* L. (hereafter *V. album*) (Fig. 1B). Harvesting took place on three occasions between May and August 2020, with care being taken to keep the attached host branch as long as possible to minimize desiccation artefacts. In addition, the acropetal ends were sealed with Parafilm (Bemis, Neenah, WI, USA) and the basipetal cut edges were placed in water buckets. An exception was the 9-year-old mistletoe used for the digital image correlation analysis, which had been harvested in January 2020; it was tested on the day after harvest and was not stored wet overnight. All samples were tested no later than 10 d after being harvested. The age of the harvested mistletoes was determined by counting the nodes of the exophyte, with the first node corresponding to 2 years and the first branching to 4 years (Fig. 1C) (Nierhaus-Wunderwald and Lawrenz, 1997). The sex of the mistletoe was determined depending on the presence of carpellate flowers and berries (female) or staminate flowers (male). Young mistletoes (up to an age of 6 years), in which these characteristics had not yet been clearly developed, were classified as juvenile. A detailed overview on all samples can be found in Supplementary Dataset S1.

### Preparation of ‘intact samples’

**Fig. 2.**
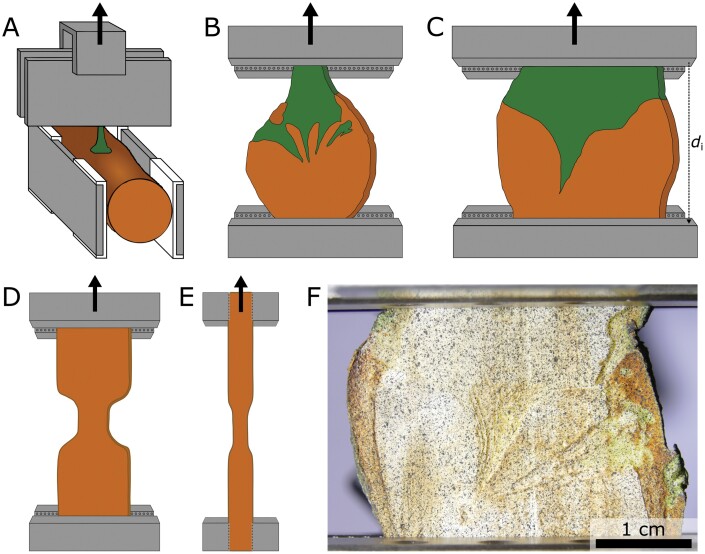
Experimental set-up of the monotonic tensile tests. (A–C) Clamping of the mistletoe–host samples for the tensile tests. (A) ‘Intact samples’: host branch (brown) and mistletoe (green) are fixed by clamps (grey) separately. Spacers (white) minimize pressure application on the middle section of the host branch, which contains the major parts of the mistletoe sinkers. (B, C) ‘Sliced samples’: host wood (brown) and mistletoe tissue (green) are fixed by clamps (grey) separately. A younger sample (B) with a higher number of smaller sinkers and an older sample (C) with one large sinker are illustrated. The initial clamp distance (*d*_i_), which is used to calculate the strain values from the displacement data, is marked as an example in (C). (D, E) Front (D) and side (E) view of the host wood prepared as dog-bone-shaped samples with reduced thickness in the narrowed section. The arrows indicate the movement direction of the upper clamps during the tensile test. (F) Sliced sample with a random speckle pattern sprayed onto the surface for digital image correlation analysis.

So-called ‘intact samples’ consisted of the hypertrophied area of the host branch and the basal part of the mistletoe exophyte (Fig. 2A). The term ‘intact’ refers to the unaffected mistletoe–host interface. Because of the limited opening range of the clamps of the testing machine, only host branches with a maximum diameter of 5 cm, aged between 3 and 13 years (Fig. 1C), were selected as intact type samples. The host branches were trimmed to a sample length of 6–14 cm, with the mistletoe centred between the sample ends. The basal mistletoe branches were trimmed to a length of 2–5 cm and all other mistletoe shoots were removed (Fig. 1B).

### Preparation of ‘sliced samples’

So-called ‘sliced samples’ were obtained by sawing cross-sections from the mistletoe–host interaction site. The resulting samples consisted of mistletoe sinkers in the upper part and host wood in the lower part (Fig. 2B, C). Subsequently, they were smoothed to an even thickness of 5–8 mm with sandpaper (LUX Universal-Schleifpapier K120, Emil Lux GmbH & Co. KG, Wermelskirchen, Germany). The sliced mistletoes were between 6 and 21 years old (Fig. 1C; including the oldest mistletoe found on the host tree). Samples were chosen so that they were large enough to allow the clamping of the mistletoe without simultaneous fixing of the host wood tissue with the same clamp.

### Clamping of ‘intact samples’

Intact samples were clamped by fixing the host branch with the lower clamp and the basal mistletoe branch with the upper clamp (Fig. 2A). Very thin mistletoe branches with a diameter of 3 mm or less were protected from being crushed by the clamps by placing them between two grooved metal plates with a diameter between 0.5 and 2.5 mm. The longitudinal axis of the host branch was oriented normal to the tensile axis (Fig. 2A). Ideally, the clamped mistletoe branch should be oriented exactly normal to this axis. However, the mistletoes did not usually grow exactly perpendicularly to the host branch axis and therefore may have (slightly) deviated from the tensile direction. Spacers were placed between the clamps and the host branch, but making sure that pressure was mainly applied to the ends of the host branch section in order to prevent preloads of the centrally positioned mistletoe sinkers because of clamping. The spacers were designed in SolidWorks (Education Edition 2017 SP5.0, Dassault Sytèmes SolidWorks Corp., Vélizy-Villacoublay, France) and 3D-printed (Objet260 Connex3, Stratasys, Eden Prairie, MI, USA) using RGD450 for the spacers and SUP706 as the supporting material (both from Alphacam GmbH, Schorndorf, Germany). These spacers inserted an additional distance of 0.5–1.5 cm between the branch and the clamp. The surface of the spacers was rough to prevent slippage of the samples. Any indication of early damage of the sinkers because of clamping led to the exclusion of the sample from analyses.

### Clamping of ‘sliced samples’

We selected the mistletoe samples in such a way that only the mistletoe tissue was fixed in the upper clamp of the testing machine and only host wood was fixed in the lower clamp (Fig. 2B, C).

### Preparation of host wood samples

We prepared dog-bone-shaped samples of plain host wood (from uninfected branches of the infested *A. flava* host tree) that were cut in longitudinal (tested parallel to the wood grain) and tangential (tested normal to the wood grain) directions (Fig. 2D, E). A maximum of two samples per orientation were cut from five selected branches. The section thickness ranged between 4.0 and 8.5 mm, the width between 13.0 and 18.0 mm, and the length was at least 30.0 mm. The tapered region was between 4.0 and 9.9 mm wide. For the longitudinal samples, not only the width, but also the thickness (between 2 and 4 mm) of the sample was slightly tapered (Fig. 2E).

### Tensile testing

An Inspekt Retrofit universal testing machine (Hegewald & Peschke, Nossen, Germany) equipped with a 10 kN load cell was used for all tensile tests. Prior to the test, the distance between the upper and lower clamps was measured as the initial distance *d*_i_. The tensile speed (cross-head displacement) was set at 0.1 mm s^−1^ and the test stopped after sample failure, with force and displacement data being recorded at 50 Hz. The data were rejected if any slippage of the sample could be detected during the test. A total of 39 intact and 31 sliced mistletoe–host samples, plus nine tangential and five longitudinal host wood samples were used for further data evaluation. In addition to the categorization according to the type of preparation (‘intact samples’ and ‘sliced samples’), a distinction between the mistletoe–host samples was also made as to whether the fracture ran at least partially along one of the clamping jaws (‘clamp failure’) or whether the fracture occurred only in the area between the clamping jaws (‘interface failure’). Two samples failed only within the mistletoe tissue; however, they were also assigned to the ‘interface failure’ group, because the interface properties were expected to have a substantial influence on mechanical behaviour. Thus, the samples were grouped as follows: ‘intact samples with interface failure’ (*n*=20), ‘intact samples with clamp failure’ (*n*=19), ‘sliced samples with interface failure’ (*n*=13), and ‘sliced samples with clamp failure’ (*n*=18).

### Digital image correlation

A random speckle pattern was applied to the front surface of four sliced samples by using black spray paint (Liquitex, Cincinnati, OH, USA) in order to measure local surface strains during tensile loading by the digital image correlation (DIC) technique (Fig. 2F). The speckled surface was recorded during monotonic loading with a Basler ace camera (acA2040-90um) and the corresponding pylon Viewer software (version 5.0.5.8999, both Basler AG, Ahrensburg, Germany), with a 35 mm lens (LM35HC, Kowa Optimed Deutschland GmbH, Düsseldorf, Germany), an exposure time of 1000 µs, and an acquisition frame rate of 50 fps. For additional illumination of the sample, we used a high-performance LED light source (Veritas Constellation 120 W, Pasadena, CA, USA).

DIC was applied to the image stack of the tensile tests by using Aramis (version 2016 Professional, GOM GmbH, Braunschweig, Germany) software. A surface component of the random surface pattern with a facet size of 20 pixels and a maximum point distance of 12 pixels was employed, with an image of the unloaded stage as reference. Throughout the tensile test, major strains (strain in the direction of the highest deformation) were calculated over the entire surface and along a section through the sinker and host wood perpendicular to the tensile load. For one of the four samples (9-year-old mistletoe), the major strain was also measured at three representative points in the centre of the sinker, at the site of first crack initiation, and at the tip of the sinker that remained in the host wood after sample failure.

### Fractured surface analysis

**Fig. 3.**
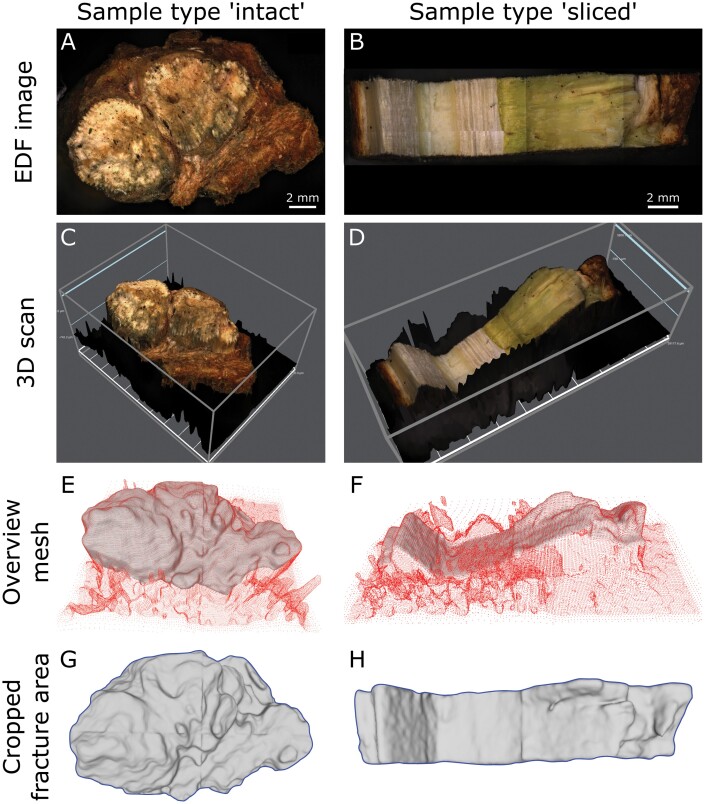
Determination of the rough fractured surface by using digital microscopy. The stepwise analysis of the fractured surface is exemplarily shown for a sample type ‘intact’ (A, C, E, G) and ‘sliced’ (B, D, F, H). Images of the fractured samples were captured with an extended depth of field (EDF) (A, B). These images provide good differentiation, especially for the sliced type samples, between the host wood tissue (light brown) and the mistletoe tissue (green). 3D scans of the fracture areas were captured (C, D), with the background appearing in black. These scans were converted to point clouds (red), in which the fracture area (grey) was determined by hand (E, F). Meshlab software was used to calculate the rough fracture area (*A*_r_) from the resulting topographic models (grey) (G, H). To determine the corresponding projected fracture surface (*A*_c_), the height information of the model was removed, and the resulting area was calculated (corresponding to the area of the blue shape).

The fractured surface of all tested samples (for one of the resulting halves) was scanned with a Smartzoom 5 digital microscope and the corresponding Zen2.6 pro software (version 6.1.7601, both Carl Zeiss AG, Oberkochen, Germany) (Fig. 3A–D). Most samples were scanned at ×34 zoom, whereas ×70 or ×100 zoom was applied for small samples. This resulted in scanning point densities of 0.007, 0.003, and 0.002 mm, respectively. The increment of scans along the *z*-axis was 50 µm for smooth surfaces or 100 µm for samples with distinct height differences along the fracture. The resulting 3D data were saved as coordinates of a point cloud (.txt files) and loaded into MeshLab (version v2020.07, Cignoni *et al.*, 2008) (Fig. 3E, F). Surface normals were computed for the point sets with a neighbour number of 10 and a smooth iteration of 0, and the surface was reconstructed with a screened Poisson algorithm (Kazhdan and Hoppe, 2013). The rough surface was manually trimmed to discard non-fractured parts and to keep only the relevant area. The relevant area was measured using the ‘Compute Geometric Measures’ function and defined as *A*_r_ (Fig. 3G, H). Additionally, the corresponding projected fractured area *A*_c_ was measured based on the two-dimensional images that lacked height information by using ImageJ (Fiji) software (version 1.52p; Schindelin *et al.*, 2012).

Samples were either scanned 1–5 d after being tested and were kept humid during this time by being wrapped in wet cloths and being stored in a refrigerator at 5 °C (42 host–mistletoe samples and all 14 host wood samples) or stored dry and rehydrated for scanning (28 host–mistletoe samples). For rehydration, samples were immersed in water for 2 s, wrapped in wet clothes and stored in a refrigerator at 5 °C overnight. In order to determine the influence of rehydration, the rough fracture surface for 10 freshly measured samples was additionally measured in the rehydrated state and their ratios were calculated (*A*_fresh_/*A*_rehydrated_). All fresh and rehydrated samples had a median ratio of 0.997 and an interquartile range of 0.066 and were therefore pooled for this study.

### Calculation of mechanical properties

All calculations were performed with programming language R (version 4.0, R Core Team, 2020) using RStudio (version 1.1.442; RStudio Team, 2016). Force data were adjusted by using values after failure as tare values (the largest possible data range with a standard deviation of less than 0.005 was used). The maximum force *F*_max_ was determined based on force–displacement data. Tensile strength was calculated as


$$\begin{array}{l} \sigma_{max}\;\;\;=\;\frac{F_{max}}{A} \end{array}$$ (1)

For the fractured surface *A*, either the values of the rough fractured area (*A*_r_) or those of the corresponding projected area (*A*_c_) were used for calculations, resulting in the ultimate tensile strength values σ_max(r)_ and σ_max(c)_, respectively. Work of fracture *W* was calculated as the trapezoidal numerical integral under the force–displacement curve. To calculate the deformation energy *E*_f_, work was normalized over the failure area, distinguishing again between *A*_r_ and *A*_c_:


$$\begin{array}{l} E_{fr}\;=\;\frac{W}{A_{r}} \end{array}$$ (2)

$$\begin{array}{l} E_{fc}\;=\;\frac{W}{A_{c}} \end{array}$$ (3)

The initial clamping jaw distance *d*_i_ and the displacement data were used to calculate the strain values. The deformation at break was defined as the strain at maximum stress. The roughness *R* of the fractured area was measured as:


$$\begin{array}{l} R\;=\;\frac{A_{r}}{A_{c}} \end{array}$$ (4)

The axial rigidity *k* (a more reliable measure for fracture tests than the axial stiffness, which does not take into account the length of the sample) was calculated as:


$$\begin{array}{l} k\;=E\;\times \frac{A}{d_{i}} \end{array}$$ (5)

with Young’s modulus *E*, the cross-sectional area *A* and the initial clamping jaw distance *d*_i_. *E*×*A* was determined as the slope of the linear-elastic region in the force–displacement diagram by using the lm() function in R. The initial linear slope of the main force peak was selected for calculation, whereas smaller peaks before the main peak were ignored.

### Statistical analyses

The R packages ‘car’ (Fox and Weisberg, 2019), ‘nortest’, ‘tidyr’, ‘ggplot2’ (Wickham, 2016), ‘gridExtra’, ‘ggsignif’, and ‘dplyr’ were used for statistical analyses and data visualization. Samples of the same test preparation (‘intact’ or ‘sliced’) were pooled for a mechanical variable if no significant difference was found between fracture type (‘clamp’ and ‘interface’) and if no correlation with age was found for these groups (*P*<0.05). The data of the groups were tested for the normal distribution (Shapiro–Wilk test) and homogeneity of variances (Levene’s test).

For the statistical test of the dependence on mistletoe sex, no distinction was made between the fracture types. Only the age-independent variables were statistically tested for sex dependence (roughness, tensile strength, fracture energy, deformation at break, and axial rigidity). For the intact samples, female, male, and juvenile mistletoe samples were compared, whereas for the sliced samples, comparisons were made only between female and male mistletoe samples, as no juvenile samples were present in this group. Only the age-independent variables were tested for their dependence on sex. Because of the small sample size of the data, the Wilcoxon rank-sum test (two-sided) was used for all sex comparisons. To test for correlation with age, Pearson’s test was used for groups with normally distributed data and Spearman’s test for groups with non-normally distributed data. If the pooling of the failure groups was successful, the same procedure was used to test whether the pooled data showed a correlation with the age of the mistletoe.

When no correlation was found with age in any of the (sub-)groups, we tested whether significant differences could be found between the groups. If both groups were tested for normally distributed data and equality of variances, an independent two-sample Student’s *t*-test (two-sided) was used; otherwise, Wilcoxon’s rank-sum test (two-sided) was employed. An α-level of 0.05 was applied as the significance level for all statistical tests. In addition, for the group-wise comparisons, statistical significance is indicated by *P*<0.05, *P*<0.01, and *P*<0.001.

## Results

### Monotonic tensile tests

**Fig. 4.**
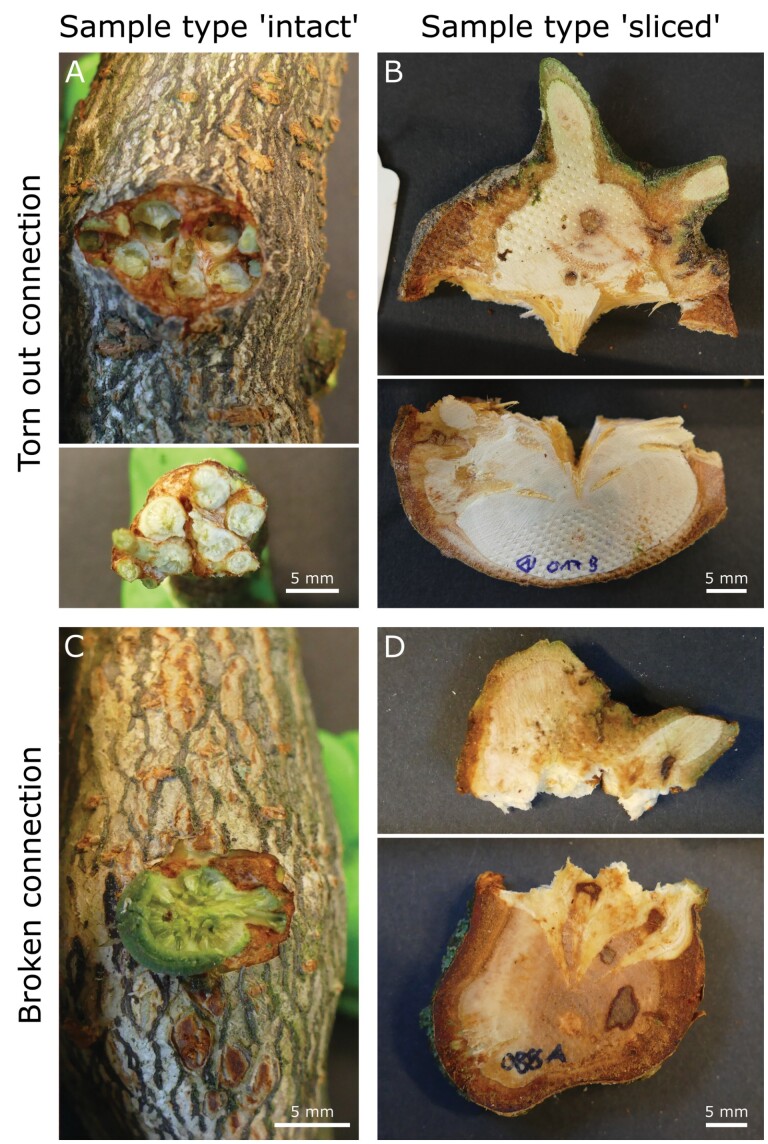
Exemplar images of torn out (A, B) and broken (C, D) mistletoe–host connections after tensile testing to failure. Both failure modes were found for intact (A, C) and sliced samples (B, D; the dot pattern in the wood of the samples indicates the position of the clamps). (A) Eight-year-old sample with several torn out sinkers. The upper image shows the host branch including the torn out hole; the lower image shows the pulled out part of the mistletoe. (B) Fourteen-year-old sample with failure along the central sinker with its tip remaining in the host (lower image). (C) Six-year-old sample with the basal branch of the mistletoe and some host bark ruptured. (D) Nine-year-old sample with failure that propagated through the mistletoe and with large parts of the sinkers remaining inside the host (lower image). The scale bars of (A, B, D) apply to both respective subimages.

From 39 intact tensile test samples, 19 samples ruptured along the clamping. For the other 20 tests, the clamping was not involved in sample failure. In nearly all of those cases, the interface between host and mistletoe was part of the fractured area. Often, the rupture appeared along the interface and tore off the sinker tips, resulting in a hole in the host tissue (‘torn out connection’; Fig. 4A, B). In some cases, the mistletoe ruptured within the transition zone between the mistletoe branch and the sinker. The sinker was still connected to the host afterwards but the mistletoe basal branch and parts of the host bark were removed (‘broken connection’; Fig. 4C, D). In two cases only, the mistletoe branch failed without involvement of host tissue. No sample ruptured only within the host wood and without additionally damaging the mistletoe tissue. For the sliced type tensile test samples, clamping was involved in sample failure in 18 out of 31 tests. For all other samples, the fracture ran at least partly along the sinker and did not involve clamped tissue. All host wood samples ruptured within the tapered region of their dog-bone shape. Samples that were tested normal to the grain mostly failed along the edge of an annual ring.

**Fig. 5.**
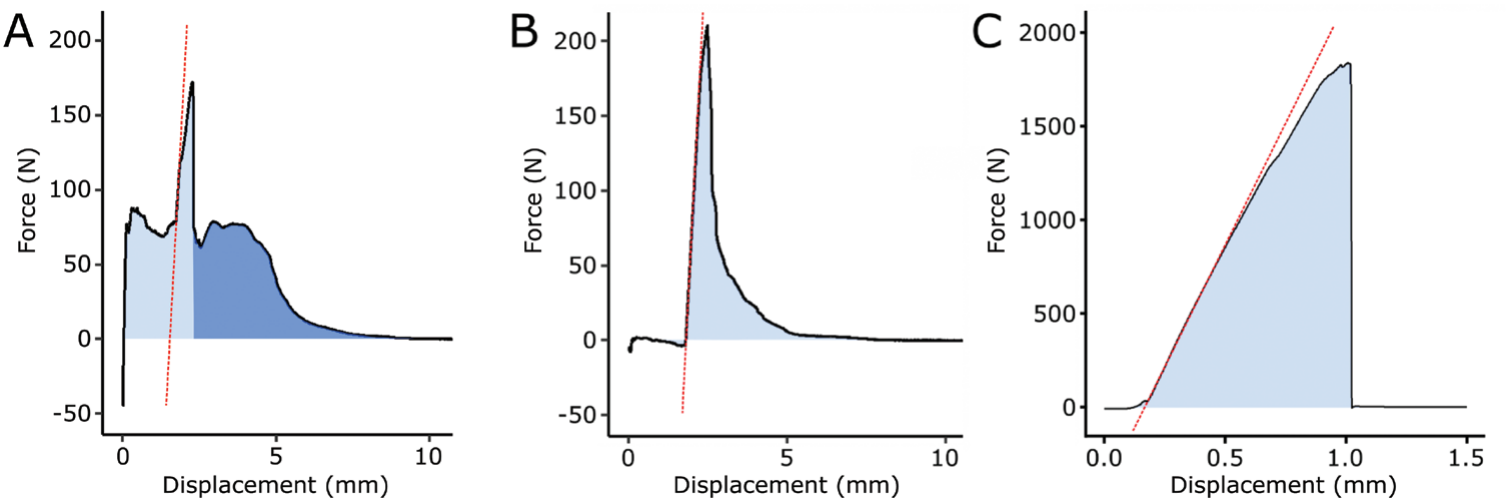
Force–displacement graphs of the tensile tests. Representative curves are shown for an intact type mistletoe–host sample (A), for a sliced type mistletoe–host sample (B), and for a host wood sample (C). The slope of the linear part of the main peak (red dotted line; used for calculation of axial rigidity) and the integral of the curve (blue; indicating work of fracture, which was used to calculate deformation energy) are marked. For the intact type sample (A), a distinction is made between pre- (light blue) and post-failure (dark blue) regions.

Initial negative values were apparent in some of the intact type samples after data adjustment (Fig. 5A) but these were normalized immediately after the start of the test. In the sliced type samples, this effect was not or only very slightly noticeable (Fig. 5B), which led us to assume that they were caused by compression during the clamping of the intact samples. For the intact type samples, one or more smaller peaks (at about 90 N in Fig. 5A) before the main force peak (at about 170 N in Fig. 5A) were observed (pre-failure). After sample failure, the force values tended not to drop to 0 directly but to form smaller trailing peaks or plateaus (post-failure) (at about 80 N in Fig. 5A). The force values of the sliced type samples increased monotonically, forming one major peak with a readily distinguishable linear-elastic region and a fast drop after sample failure (Fig. 5B). Force graphs of the host wood samples were similar to those of the sliced type samples, with a more abrupt drop in force after failure (Fig. 5C). Datasets with maximum forces smaller than 10 N exhibited noise in the force data but were still included in the evaluation because the signal-to-noise ratio was large enough to obtain meaningful values.

### Sex-dependency

Twenty-six of the intact type samples were female, eight male, and five juvenile. In the sliced type samples, we tested no juvenile mistletoes, but 25 female and six male ones. No significant difference was found between female and male samples for any of the variables tested, within either the intact or the sliced type samples. Significant differences between juvenile and female samples (*P*=0.016) and juvenile and male samples (*P*=0.011) were only found for axial rigidity. Since no influence of sex and only a small influence between juvenile and mature samples was found, all samples were pooled for further analysis. Detailed correlation statistics on mistletoe sex can be found in Supplementary Table S1.

### Age-dependent variables

**Fig. 6.**
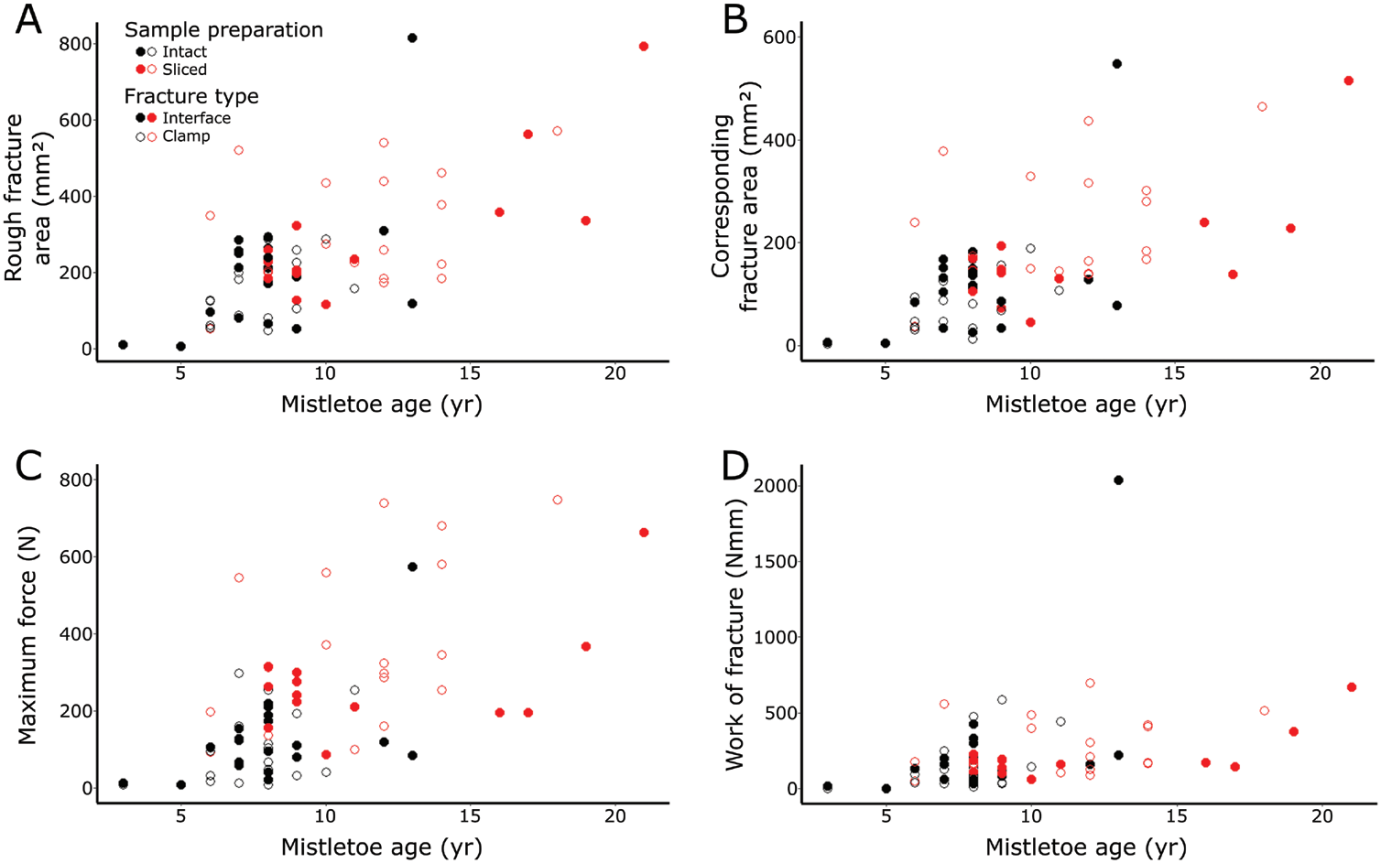
Scatter plots of the age-dependent variables. The rough fracture area (A), the corresponding fracture area (B), the maximum force (C), and the work of fracture (D) are plotted against the age of the mistletoe samples. All four variables show a significant correlation with age in at least one subgroup (intact clamp (*P*=0.010) and section interface (*P*=0.027) for rough fracture area; intact clamp (*P*=0.004) for corresponding fracture area; section clamp (*P*=0.033) for maximum force; intact interface (*P*=0.039) for work; Supplementary Table S3). Data points for ‘intact’ type samples are shown in black and for ‘sliced’ type samples in red. Filled circles indicate measurements with a failure at the mistletoe–host interface, open circles show measurements where failure occurred at least partly along the clamp.

The rough fractured area ranged between 7.9 and 815.5 mm² (Fig. 6A), with younger samples tending to have smaller values. We found a positive correlation of rough fractured area with age for the intact type samples with clamp failure (Pearson *r=*0.58, *P=*0.010) and for the sliced type samples with interface failure (Spearman *r=*0.61, *P=*0.027). Values for the corresponding projected fracture area, where the height information of the surface was neglected, ranged between 2.4 and 515.7 mm² (Fig. 6B), with a positive correlation with age for the sliced type samples with clamp failure (Pearson *r=*0.62, *P=*0.004). Maximum force values of mistletoe–host samples ranged between 6.9 and 747.6 N (Fig. 6C), with a positive correlation with age for the sliced type samples with clamp failure (Pearson *r=*0.50, *P=*0.033). Values for work ranged between 0.004 and 2.035 N m (Fig. 6D), showing a positive correlation with age for the intact type samples with interface failure (Spearman *r=*0.46, *P=*0.039). Since these four variables showed a correlation with age for at least one of the four subgroups, they were not included in further statistical tests. Values for tensile strength, deformation energy, deformation at break, fracture surface roughness, and axial rigidity showed no significant correlation with age in any of the subgroups. Detailed correlation statistics can be found in Supplementary Table S2.

In a comparison of the host wood samples with different tensile direction, the values for roughness, tensile strength (for both rough and corresponding projected surface), deformation energy (for both rough and corresponding projected surface), and axial rigidity were significantly larger for samples tested parallel to the wood grain (all *P*<0.001), whereas the values for deformation at break were significantly higher for the samples tested normal to the wood grain (*P*<0.01; detailed statistical host wood analyses can be found in Supplementary Table S3). For further comparisons between the host wood and the mistletoe–host interface, only the wood samples tested normal to the grain were considered, as this corresponded to the direction of loading, both for intact and sliced type mistletoe–host samples.

### Age-independent variables

The median fractured roughness of mistletoe–host samples was about 21.8–64.5% higher (*P*<0.001) compared with the host wood samples (Table 1; detailed statistical analyses can be found in Supplementary Table S2). Sliced type samples that ruptured (at least partly) along the clamp showed a 17.2% lower median roughness compared with sliced type samples that failed entirely within the sample (*P*<0.01) and a 26.9–35.1% lower median roughness than the intact type samples (*P*<0.001). No significant difference was found between the two failure type subgroups of the intact type samples. For deformation at break, values did not differ significantly between intact type samples and host samples. The median deformation at break value for the intact type samples that failed at the interface was 172.1% (*P*<0.001) higher than those of the sliced type samples that failed at the interface, and 91.8% (*P*<0.01) higher than those of the sliced type samples that failed (partly) at the clamp. Sliced type samples that failed at the interface had a 59.4% lower median deformation at break than the host wood samples.

**Table 1.** Overview of the age-independent mechanical properties of the mistletoe–host interface (*Viscum album*) and the host wood (*Aesculus flava*)

Samples	Mistletoe-host				Host	
Preparation	Intact (*n*=39)		Sliced (*n*=31)			
Failure mode/Orientation	Interface (*n*=20)	Clamp (*n*=19)	Interface (*n*=13)	Clamp (*n*=18)	Normal to grain (*n*=9)	Parallel to grain (*n*=5)
Roughness	1.70 (0.41)^A^	1.81 (0.87)^A^	1.57 (0.74)^A^	1.34 (0.22)^B^	1.10 (0.05)^C^	3.12 (1.67)
Tensile strength (rough surface) (MPa)	0.66 (0.47)^A^		1.14 (0.78)^B^		3.43 (0.65)^C^	19.62 (9.16)
Tensile strength (corresponding projected surface) (MPa)	1.24 (1.65)^A^		1.61 (0.63)^B^		3.78 (0.42)^C^	58.94 (19.69)
Fracture energy (rough surface) (J m^−^²)	0.81 (0.94)^A^		0.79 (0.40)^A^		1.50 (0.44)^C^	7.39 (5.70)
Fracture energy (corresponding projected surface) (J m^−^²)	1.62 (1.25)^A^		1.11 (0.54)^B^		1.65 (0.40)^A^	18.35 (15.10)
Deformation at break (%)	11.7 (5.6)^A^	7.4 (7.7)^AB^	4.3 (2.2)^B^	6.1 (2.1)^BC^	10.6 (6.0)^AC^	4.4 (0.3)
Axial rigidity (kN)	3.86 (4.46)^A^		11.21 (8.30)^B^		4.27 (2.68)^A^	58.55 (4.11)

Values are medians (interquartile range). Mistletoe–host data are subdivided according to preparation (intact versus sliced type samples) and fracture type (only at the interface versus partly at the clamp). The latter values were pooled if no significant differences were found between the two subgroups. Host wood samples are subdivided by the orientation of their sections (tangential versus longitudinal). Ultimate tensile strength (UTS) and deformation energy were calculated by using either the rough fracture area or the corresponding projected area. Uppercase letters for each row indicate the groups without statistically significant differences for the respective variable. For the statistical comparison with the host wood, only the samples tested normal to the grain were included, as their fibre orientation was similar to those of the mistletoe–host testing.

The values for ultimate tensile strength (σ_max(r)_ and σ_max(c)_), deformation energy (*E*_fr_ and *E*_fc_) and axial rigidity (*k*) did not differ significantly between the samples ruptured (partly) along the clamp or only at the interface, enabling us to pool these groups. Compared with the host wood samples, the median tensile strength calculated with the rough surface was reduced by 66.8% for the sliced type mistletoe–host samples (*P*<0.001) and by 80.8% for the intact type samples (*P*<0.001) (Table 1). The values for the sliced type samples were 72.7% higher compared with intact type samples (*P*<0.001). Results for tensile strength calculated with the corresponding projected fracture area showed similar tendencies, with intact type samples having a 67.2% lower median value (*P*<0.001) and sliced type samples having a 57.4% lower median value (*P*<0.001) than host wood. The median values of the sliced type samples were 29.8% higher than those of the intact type samples (*P*<0.05). Deformation energy calculated with the rough fractured surface was reduced by 46.0% for intact type samples compared with host wood (*P*<0.05), and by 47.3% for sliced type samples (*P*<0.001). Intact and sliced type samples did not differ significantly. For the deformation energy calculated with the corresponding projected fracture area, the intact type and the host wood samples showed no significant difference. The sliced type samples had a 31.5% (*P*<0.05) lower median value than the intact type samples, and a 32.7% (*P*<0.01) lower median value than the host samples. No significant difference was found between the host and the intact type samples for axial rigidity. Median values of the sliced type samples were 190.4% higher than intact type samples (*P*<0.001), and 162.5% higher than host wood samples (*P*<0.001).

### Digital image correlation

**Fig. 7.**
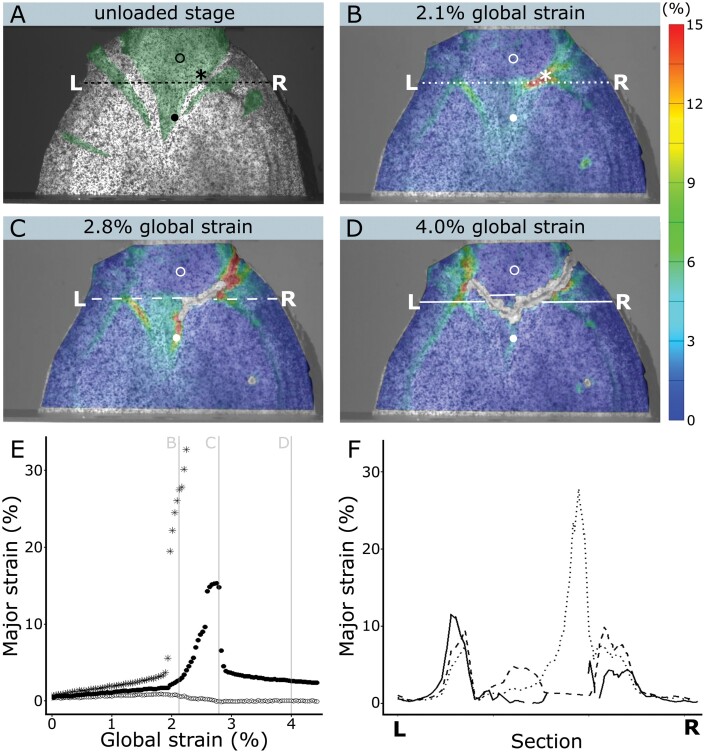
Local strain analysis of a sliced mistletoe–host sample (9-year-old mistletoe) during tensile loading by using the digital image correlation technique. (A) Unloaded stage with the mistletoe sinkers coloured in transparent green. The analysed points are marked by an open circle (within the mistletoe sinker), by an asterisk (at the site of first crack initialization), and by a filled circle (within the tip of the sinker that remains within the host wood after failure). The dashed line represents the evaluated section, with the letters L and R marking its ends. (B–D) Surface strain analyses at three different strain levels of the entire sample (global strain). Die colours indicate major strains from 0% (blue) to more than 15% (red), with the scale applying for all subfigures. The single points and the section are marked if they could be calculated for the respective stage. (B) Stage with 2.1% global strain, just before first cracks appear. (C) Stage at 2.8% global strain after first cracks are visible at the mistletoe–host interface. (D) Stage at 4.0% global strain after the upper part of the mistletoe endophyte is almost completely torn away from the host wood. (E) Major strain values for the three individual points analysed, with their form (circle, asterisk, and filled circle) corresponding to those marked in (A–D), plotted over the strain of the entire sample during tensile loading. The strains of the presented snapshots (B–D) are marked with a grey line. (F) Major strain values for the section through the sinkers at the three presented stages of 2.1% global strain (dotted line), of 2.8% global strain (dashed line) and of 4.0% global strain (solid line). The *x*-axis corresponds to the length along the section from L to R.

**Fig. 8.**
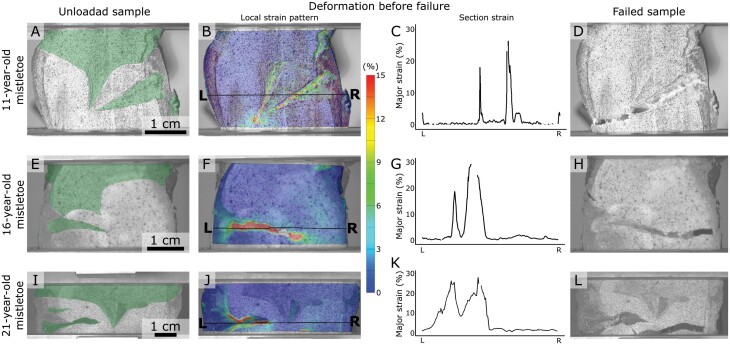
Overview of the digital image correlation analyses of an 11-year-old (A–D), a 16-year-old (E–H) and a 21-year-old (I–L) sliced mistletoe sample. (A, E, I) Unloaded sliced mistletoe–host samples (mistletoe sinkers coloured in transparent green), sprayed with a random speckle pattern. Selected stage directly before the first crack becomes visible is presented for all three samples with the corresponding local strain pattern (B, F, I) and the major strain graph of the marked section (from L to R) (C, G, K). (D, H, L) Samples after failure of the mistletoe–host interface.

The digital image correlation method was used on four selected sliced samples to determine the local strains during tensile loading. Surface detection was successfully applied for all samples over the entire area between the clamping jaws (Figs 7, 8). A loss of surface detection only occurred after the development of cracks (e.g. Fig. 7C). A single-spot analysis of the sinker (circle in Fig. 7) showed that local major strains within the sinker were below 1%, with a smooth increase during loading and a decrease after sample failure. The largest major strain rates were detected along the interfaces between the mistletoe endophyte and the host wood. Analysis of a single point at the site where the first crack initiated (asterisk in Fig. 7) showed that the main deformation under tensile load first increased slightly up to about 4% major strain, before a steep increase to over 30% was found. After the subsequent formation of cracks in this area, surface detection was no longer possible. Major strain rates for the single point within the tip of the sinker (filled circle in Fig. 7) showed a comparable first increase of up to about 2%. After cracks had formed in the upper part of the endophyte, the strain values continued to increase to a value of about 15%. After failure of the upper part of the sinker, the values dropped continuously to about 3%, indicating a plastic deformation of the mistletoe tissues close to the interaction site.

A section through the mistletoe–host interface (line from L to R in Fig. 7) revealed that almost no strain was present within the host wood throughout the tensile test. In addition, the strain peaks at the interface were found to be highly localized, as indicated by the sharp peaks in the corresponding graph (Fig. 7F). These curves also revealed that plastic deformation near the interface was capable of reaching values up to 10%. The local strain distribution throughout the tensile test is shown in Supplementary Video S1.

A comparison of the 11-, 16-, and 21-year-old mistletoe indicated that the pattern of local major strains was consistent for all samples, with very low strains in the host tissue, relatively low strains within the mistletoe and host wood tissues, and the highest values at their interface (Fig. 8). The closer the interface was to a normal orientation to the tensile direction, the earlier local strains were found and the larger they appeared. For all three samples, local major strains of about 30% were found at the sites at which the first crack formation was observed (Fig. 8C, G, K). However, the crack did not propagate exactly along the interface but mostly ran horizontally through the host wood (Fig. 8D, H, L).

## Discussion

The European mistletoe, *Viscum album*, benefits from the connection to its host branch by drawing water and mineral salts from the vascular system of the host (Heide-Jørgensen, 2008) and by having better access to sunlight because of its exposed location on the host branches with reduced investment in its own supporting material. However, this dependence on water and nutrient supplies from the host also requires continuous and proper functioning of the system. Thus, it is essential for the survival of an individual mistletoe that, after its initial anchorage, the structural integrity of the parasite–host interface is maintained during host growth throughout the lifetime of both host and mistletoe and that the functional integrity of the water supply does not break down, even under (external) mechanical stress. Mechanical loads originate not only from the often large and heavy mistletoe bushes themselves, but also from additional loads such as wind, snow, or perching birds. Some books (e.g. Heide-Jørgensen, 2008), reviews (e.g. Teixeira-Costa, 2021), and scientific articles (e.g. Teixeira-Costa and Ceccantini, 2016) can be found on the morphology and anatomy of various mistletoes and their hosts, but during our literature search, we have discovered no studies on the mechanics of the mistletoe–host connection, although abundant information is available on the distribution and ecology of *V. album* and its subspecies (e.g. Zuber, 2004).

### Morphology and anatomy of the mistletoe–host interface

Mylo *et al.* (2021) have carried out micro-computed tomography scans that provide a three-dimensional representation of the endophyte of *V. album* ssp. *album* in its host tree *A. flava*. A 4-year-old sample, representing young mistletoes, has several small sinkers, all about the same size. Even at this early stage, the endophyte extends over more than 5 cm along the host branch. The exophyte is connected to some sinkers directly and to others indirectly via cortical strands. Thus, the mechanical load imposed by the young exophyte is distributed evenly over a larger area to the sinkers and the cortical strands. With age, the individual sinkers become increasingly thick and gradually grow together. The scan of a 17-year-old sample, as a representative of older mistletoes, revealed a single but widely radiating wedge-shaped sinker. This massive endophyte bears the entire mechanical load of the old exophyte (see Fig. 2B, C for an exemplary drawing of a younger and an older endophyte).

The anatomy of the host–mistletoe interface plays a crucial role in ensuring functional integrity, namely that the mistletoe is supplied with water and nutrients by the host. Particularly challenging is the synchronous growth of the wedge-shaped sinkers and the formation of new growth rings of the host wood. Mylo *et al.* (2021) have shown that vessel elements of the mistletoe run along its longitudinal axis toward the tip and bend laterally to the host wood, which forms swirls and clusters of vessels and fibres and aligns with the mistletoe interface. This cross-grained wood near mistletoe–host interfaces, also described by Teixeira-Costa and Ceccantini (2016), allows for direct contact between the water-conducting cells of the two plants and reveals the internal stresses between these growing and competing organisms.

### Gradients avoid crack initiation

From a mechanical viewpoint, the transition between the fully lignified host wood and the non-lignified mistletoe parenchyma is prone to damage initiation because of the large differences in their mechanical properties. Mylo *et al.* (2021) have shown that the abrupt transition is smoothed by lignified mistletoe tissue directly at the interface. A gradient of lignified tissues is formed together with the bands of vessels splitting near the host interface. Lignification of the mistletoe parenchyma is found primarily in older sinkers (Smith and Gledhill, 1983), beginning at the interface of the sinker and continuing toward the centre. In general, the formation of superimposed multiple gradients, such as the hierarchically organized structural characteristics (e.g. arrangement, distribution, dimensions, and orientations of cells and cell compartments) and chemical composition (e.g. lignin), is a ‘mechanical concept’ often found in plants (Liu *et al.*, 2017). In particular, when replacing abrupt changes in material properties by a gradient, stress differences are less severe and cracking becomes less likely (Speck and Burgert, 2011). An example is the Mexican fan palm (*Washingtonia robusta*), which achieves a rigidity gradient between the stiff vascular bundles and the more flexible parenchyma by increased lignification and cell wall thickness towards the bundles (Rüggeberg *et al.*, 2008). Given lignin is a phenolic polymer of great importance for the mechanical properties of the plant cell wall (Weng and Chapple, 2010), the formation of a lignin gradient at the mistletoe–host interface should retard crack initiation and delamination under mechanical loading. Observations on tested samples that did not fail exactly along the interface, but slightly parallel to it, support this hypothesis.

### Intact and sliced samples under tensile loading

Because of their different preparation methods, intact and sliced samples have different geometries and proportions of endophyte and host wood that affect the results of their behaviour under tensile loading. Intact samples contain the unmodified endophytic system in the host wood and the basal branch of the exophyte (Fig. 2A). A perfectly normal tensile direction to the host branch axis could not be achieved for most of our samples, because of crooked mistletoe growth and extreme hypertrophy of the host branch. Therefore, the measured force values of intact type samples probably result not only from tension, but (to a limited degree) also from multiaxial effects of shear, torsion, and bending. Sliced samples only contain parts of the endophyte (Fig. 2B, C) but can be clamped more easily and, thus, the normal tensile direction to the host branch axis can be guaranteed. Additionally, this enables the study of older mistletoe samples that cannot be clamped in the tensile testing system because of increasing hypertrophy. Influences from torsional and bending stresses can be neglected for the sliced samples. However, in intact and sliced type samples, shear forces may occur at the interface as a result of the inclination angle of the mistletoe–host interface compared with the tensile direction.

### Analyses of local deformation and deformation at break

Since the mistletoe–host interface is the critical zone that ensures water supply for the mistletoe, we analysed intact and sliced samples under tensile loading up to failure. Selected monotonic tension experiments of the sliced samples were combined with DIC analysis (Fig. 2F), a procedure that offers the quantification of full-field surface deformation with high temporal resolution and (semi-)automated evaluation. This method has proven to be a valuable tool for the evaluation of two-dimensional and three-dimensional recordings (from time lapse to high speed) of plant deformation and movement, thereby forming the basis of further finite element method simulations or providing a comparison with biomimetically inspired materials systems (Mylo *et al.*, 2018; Correa *et al.*, 2020; Sachse *et al.*, 2020). When using DIC for tensile tests with flat samples, a one-camera set-up is sufficient, as all deformations take place in a single plane (Sutton *et al.*, 2008). Under tensile load, local major strains of up to 30% mainly occur close to the interfaces between the host wood and the mistletoe endophyte, before cracks develop in this region (Fig. 7). The comparison between the four samples of the different age groups suggests that this maximum strain value is not dependent on the age of the mistletoe (Fig. 8). Because of the lignin gradient between the endophytic system and the host wood described above (Mylo *et al.*, 2021), the deformations and cracks often shift slightly into the mistletoe tissue, resulting in mistletoe remains in the tear out hole (Fig. 4B). In particular, sinker–host interfaces oriented normal to the pulling direction are prone to failure. This might result in individual, horizontally oriented sinkers delaminating from the host wood under tensile load, with the more vertically oriented sinkers retaining their structural and functional integrity. Cracks propagating exclusively through the host wood were not observed in our experiments, indicating that it has a higher toughness than samples that include a mistletoe–host interface. Within the mistletoe sinkers, larger local deformations are found under tensile loading than in the host wood, and the DIC analysis after failure of the samples reveals that they undergo plastic deformation in contrast to the host wood, where almost exclusively elastic deformation is found (Fig. 7D).

Deformation at break is significantly higher for intact type samples that failed at the interface, with a median of 11.7%, than for sliced type samples, with a median value of 4.3% (*P*<0.0001; Table 1). This discrepancy indicates that the entire endophytic system, including the numerous sinkers and the cortical strands that grow in all directions and that are integrated into the host tissue, can withstand much higher strains without failure than local strains of individual regions measured from the sliced type samples. Crack formation of one sinker or cortical strand does not inevitably result in failure of the entire endophytic system, because the other structures securely anchor the mistletoe in its host. In other words, the risk of total failure is reduced by distributing the loads over several elements. The concept of load sharing is widespread in biology and can be found, for example, in hierarchically structured anchoring systems such as root systems (Stubbs *et al.*, 2019) and tendrils (Steinbrecher *et al.*, 2010; Rowe and Speck, 2015). In contrast to intact samples, many endophytic structures are absent from the sliced samples, as they consist mainly of host wood and (parts of) the mistletoe sinkers that are close to the penetration site (Fig. 4). Sinkers run primarily in the radial direction of the host branch leading to sliced samples whose tissue composition differs markedly in a tangential direction but not in a longitudinal direction (orientations with respect to the host branch). Because of these geometric conditions, cracks run rapidly through the entire sample and lead to failure.

### Analyses of the roughness of fractured surfaces

The fractures of the samples do not form straight fracture lines. Instead, the tissues fracture unevenly in the different layers and cracks occasionally change direction. This type of cracking leads to the formation of a rough fractured surface, which we have been able to quantify using digital microscopy (Fig. 3). A completely smooth fractured surface has a nominal value of 1.0, whereas higher values represent increased roughness. Median roughness values of the mistletoe–host samples were 1.70 and 1.81 for intact type samples and 1.34 and 1.57 for sliced type samples, with regard to the interface failure and clamp failure samples, respectively. In comparison, transverse host samples have a median of 1.10 (Table 1), because cracks often run along the annual rings resulting in an almost smooth fracture surface. Since roughness is an important aspect in crack formation, we took it into account when calculating mechanical properties of the samples. Unlike some recent data for *Macadamia* shells (Schüler *et al.*, 2014; Bührig-Polaczek *et al.*, 2016), data on the roughness of fractured surfaces in wood (Morel et al., 1998, 2002; Thoma *et al.*, 2015) cannot be compared with our data, because the reported roughness was either not considered after mechanical tests or it was calculated differently. The latter was the case in, for example, Beismann *et al.* (2000) who calculated the relative roughness as the proportion between the area showing a rough surface and the total cross-sectional area of the samples. Some authors merely included the cross-sectional area but did not take into consideration the profile of the fractured surface in the measurements of the fractured areas (Beismann *et al.*, 2000; Kane *et al.*, 2008; Masselter *et al.*, 2011; Schwager *et al.*, 2013; Bunk *et al.*, 2019). The roughness of fracture surfaces of *Macadamia* shells was measured using a laser scanning confocal microscope focused on various regions of the shell (outer, middle, inner) and according to various compressive loading directions. The rough surface was also compared with the corresponding projected surface and exhibited a value that was larger than the projected surface by a median factor of 10–25, with the differences being caused by the sandwich structure containing varying cellular compositions (Schüler *et al.*, 2014; Bührig-Polaczek *et al.*, 2016). Compared with our analyses of the fracture areas, the *Macadamia* was scanned at a higher resolution, with a markedly smaller area of interest, and meso-, micro-, and nano-structuring was taken into consideration. In our approach, using digital microscopy, we were methodologically limited to the meso- and micro-range but had the advantage of taking into account the entire fracture surface. The values obtained allowed good comparisons to be made between the analysed samples (e.g. between the wood host samples tested parallel or normal to the grain) but could not be compared directly with other experimental data because of the different spatial resolution of the employed techniques.

### Age-dependency of the mechanics and size

The European mistletoe forms decades-long relationships with its host tree. During the four stages of development, it establishes a mechanically strong connection with its host. After germination, the hypocotyl adheres to the host via a lipidic glue and forms a holdfast with epidermal papillae that grow into the host cortex (Thoday, 1951; Heide-Jørgensen, 2008). In its first year, the mistletoe grows and spreads completely endophytically. Some host trees may respond to this invasion with an accumulation of polyphenols in the phellem tissue, lignification of the phellem, and an increase in fibre cells, which may impede the formation of a strong connection (Grundmann *et al.*, 2014). Over the years, the mistletoe grows increasingly more cortical strands and sinkers that anchor the mistletoe individually to the host and reduce the risk of failure of the entire endophyte. Throughout its lifetime and during every stage of its development from a seedling via the young mistletoe to the 20-year-old shrubs with a diameter of up to 2 m, the mistletoe requires an uninterrupted supply of water and mineral salts. Moreover, it has to adapt throughout its life to the growth of its host branch.

Given plants can show changes in mechanical properties at their various ontogeny stages (Speck and Burgert, 2011), we analysed the effect of age on the linear-elastic behaviour and fracture mechanics of intact and sliced type mistletoe–host samples that failed at the clamp or at the parasite–host interface. The samples examined were from 3- to 21-year-old mistletoes (Fig. 1). Figure 6 shows the age-dependency of the size-dependent variables of maximal force, fracture area (rough and corresponding projected), and work. Our analyses reveal furthermore that axial rigidity as a measure of the slope in the linear-elastic range under tension, tensile strength, deformation energy, deformation at break, and surface roughness, which represent the fracture behaviour, show no significant correlation with age (Supplementary Table S3). This indicates that even young mistletoes can establish a stable connection to the host that does not change significantly over time, thus ensuring a vital connection to the host from an early developmental stage. We found no influence of sex in the mature dioecious mistletoes for all the tested structural and mechanical parameters.

### Mechanics under tensile loading

To our knowledge, no studies on the mechanical properties or qualitative descriptions of the failure of the connection between a mistletoe and its host have been carried out. Instead, the focus is usually on the effects of mistletoe infestation with regard to the quality of the host wood and its further use as building material or, in cases of poor quality, for paper production (Piirto *et al.*, 1974). From our tensile tests until failure, we have been able to calculate various mechanical variables of the samples. We are aware that the employed equations (see ‘Materials and methods’) are valid only for isotropic and homogeneous materials. Nevertheless, we have used this approach to quantify the mechanical performance of our biological samples with the help of these formulae, knowing that the samples, with all the endophytic elements in the host wood, do not represent a homogeneous and isotropic material but rather an inhomogeneous and anisotropic structure.

For the calculation of the *axial rigidity* as a measure of the mechanical behaviour in the linear-elastic range, we would require values for Young’s modulus and the cross-sectional area. Since our mistletoe–host samples did not have a uniform cross-section along the sample axis, the cross-sectional area could not be determined accurately and, thus, we could not compute the elastic modulus. Instead, we calculated the axial rigidity from the slope of the linear-elastic region in the force–displacement diagram divided by the initial clamp distance. The average axial rigidity of the sliced type samples is approximately three times the median value of the intact type samples (Table 1). This difference is related to the size dependence of the axial rigidity caused by the cross-sectional area, the latter being larger for the sliced type samples because of their older age (Figs 1, 6B). Values of axial rigidity of host wood samples tested parallel to the grain and those tested normal to the grain differed significantly (*P*<0.001) as a result of the strong anisotropy and inhomogeneity of the wood. The comparison of the axial rigidity of host wood tested normal to the grain and of the intact type samples showed no significant differences. In contrast to the intact type samples, the axial rigidity of sliced type samples was three times higher than that of host wood loaded normal to the grain. This can again be explained because axial rigidity is not a size-independent variable and because the host wood samples had a smaller cross-sectional area as they were sawn into a dog-bone shape (Fig. 2D, E).

The influence of the surface roughness on the calculation of the *strength* at failure becomes evident when both the rough surface *A*_r_ and the corresponding projected area *A*_c_ are used for comparative calculations. The variable accuracy of the determination of the fracture surfaces with and without roughness is reflected in the median values of the tensile strength. For intact type samples, the tensile strength based on the corresponding projected area (without roughness) was about twice as high as the tensile strength based on the rough area (Table 1), taking into account that the rough fracture surface leads to median values of the tensile strength of 0.66 MPa for intact type samples and 1.14 MPa for sliced type samples. The comparatively low ultimate tensile strength of the intact type samples can probably be explained by the additional torsional and bending loads, which are unavoidable during the tensile tests as outlined above. Moreover, the preparation of the sliced type samples might have had an impact, because they have large surfaces prone to dehydration, which can stiffen the material. The time between cutting and testing was kept short, i.e. to approximately 15 min, and dehydration was slowed down by using wet towels. Therefore, the effect of stiffening by dehydration is considered to be relatively small. However, the difference in tensile strength can be attributed to the different roughness of the fracture surfaces of the intact and sliced type samples. Compared with the host wood samples tested normal to the grain, tensile strength was reduced by 66.8% for sliced type mistletoe–host samples and by 80.8% for intact type samples (Table 1). Host wood samples with grain aligned normal to the tensile direction were used for comparison because they were tested with the same grain orientation as the host wood in the mistletoe–host samples. We have assumed that the lower values of the mistletoe–host samples result from the decreased tensile strength of the mistletoe tissues and the weaker interface between the mistletoe and host. A comparison with literature values for the host wood is difficult. Values for *A. flava* cannot be found, and an additional obstacle is the need for data from green wood, since the wood samples in our study were fresh. However, wood of *A. flava* has similarities to the wood of *Populus*, *Tilia*, and *Liriodendron* (Bergman *et al.*, 2010). Therefore, the tensile strength of the species *Populus tremuloides*, *Tilia americana*, and *Liriodendron tulipifera* have been used for comparison with values ranging between 1.6 and 3.5 MPa for tension normal to grain. These values are in good agreement with the result for host wood samples in this study having a median of 3.4 MPa. Our host wood samples tested parallel to the grain have a median tensile strength of 19.6 MPa, which is 5.7 times higher, again illustrating the anisotropy of wood because of its fibre orientation. To determine the mechanical properties of the host wood, care was taken to ensure that only wood from non-infected branches was tested. However, Anselmo-Moreira *et al.* (2019) showed that parasitism by the mistletoe can lead to systemic effects on the host metabolism (e.g. the production of tannins), which may also have an effect on the mechanical properties of the wood of non-infested branches. In addition, Piirto *et al.* (1974) showed that an infestation of dwarf mistletoe has an effect not only on the mechanical properties (such as bending stiffness) of the infested branches but also on those of the non-infested branches. Although the force–displacement curves of the tensile tests differ between intact and sliced type samples (Fig. 5), their *deformation energy* is similar with medians of 0.79 J m^−2^ and 0.81 J m^−2^, respectively. Whereas the sliced type samples show a rapid increase in force with a single peak followed by an immediate decrease (Fig. 5B), the graphs of the intact type samples mostly have several smaller peaks around the maximal force (Fig. 5A). These additional smaller peaks increase deformation energy and delay the ultimate failure of the intact type sample. The single peaks can be assigned to the numerous sinkers that anchor the mistletoe in the host branch and that fail one after another, creating a benign multi-step sample failure. The failure of an individual sinker in an intact type sample does not lead to complete failure, but is rather preceded by several successive pre-failure events. The effect of these pre-failure events is reflected in the higher deformation at break as described above. A comparable benign failure can be found with Boston ivy and its successive (pre-)failures of the individual adhesive pads under tensile load (Steinbrecher *et al.*, 2010; Rowe and Speck, 2015). Sliced type samples, on the other hand, usually contain only a few sinkers and thus provide less individual anchorage in the host wood. Furthermore, because of the cutting of the samples, they are not completely surrounded by host wood, causing cracks to make their way through the sliced sample along the remaining mistletoe–host interface and immediately leading to brittle sample failure. Despite differences in their force–displacement curves and ultimate tensile strength values, the benign (ductile) failure mode of intact type samples and the brittle failure mode of sliced type samples result in comparable values of deformation energy. Host wood samples tested normal to the grain show a similar curve shape to sliced type samples, namely a fast increase of force, a single peak and an immediate decrease after the peak. The deformation energy of the wood samples, with a median value of 1.50 J m^−2^, is almost twice as high as that of the mistletoe–host samples (Table 1).

### Ecological aspects

Although we have carried out a purely biomechanical study of the host–mistletoe interaction, we can draw some conclusions with respect to ecological aspects. Our statements are, however, limited to the developmental stage with the conductive bridge having been established. We cannot make assumptions about the earlier stages of the haustorial initiation, the adhesive stage or the intrusive phase, because the mistletoes that we examined were between 3 and 21 years old (Fig. 1).

A close ecological connection exists between mistletoes and birds because, on the one hand, the berries are an important food source for birds and, on the other hand, birds spread the seed-containing berries on the branches of the host plant. During the course of the decades of interaction with its host, the endophytic system grows concomitantly with the host branch so that the mistletoe is always supplied with sufficient water and mineral salts from the annual growth rings of the host wood. Damage effects are mainly caused by water uptake, which can lead to drought stress in the host (Kehr, 2016). After 20 years, the richly branched, spherical mistletoe can reach a diameter of 2 m (Heide-Jørgensen, 2008) and a considerable weight. Despite the mechanical challenge of anchoring the large and heavy mistletoe, no reports of broken mistletoes without host branch failure are found in the literature or have arisen from personal observations. However, host branches may die back distally from large mistletoes (Kehr, 2016). Branches of trees with a high rate of colonization by mistletoes are particularly prone to failure, as the mistletoes require a constant water supply from the tree and represent an additional weight load. However, observations of branch failure (Weir, 1915; Kehr, 2016), but not mistletoe failure, are evidence for the robustness of the connection of parasite and host. Our results show that the functional and structural integrity of this connection is independent on the sex and age of the mistletoe and is thus guaranteed from the beginning of the successful penetration throughout the lifetime of the mistletoe. Furthermore, the mechanical failure of individual elements of the endophytic system preserves the mistletoe from sudden catastrophic failure of its entire anchorage.

## Conclusion

We investigated the mechanical properties of the interaction between the mistletoe *Viscum album* ssp. *album* and its host tree *Aesculus flava*. Intact and sliced type host–mistletoe samples, plus host wood samples for comparison, were subjected to tensile tests up to failure. By determining the fracture surface by digital microscopy, we were able to take into account the resulting surface roughness and use these values for further mechanical calculations. The results of our mechanical tests contribute to the understanding of the linear-elastic and fracture behaviour of the samples, the age- and sex-dependency of the mechanical properties, and the local surface strain distribution under tensile load:

- Mistletoe–host samples show no significant correlation with age or sex with regard to axial rigidity, ultimate tensile strength, deformation energy, deformation at break, and surface roughness.
- The tensile strength and deformation energy of mistletoe–host samples exhibit markedly smaller values compared with those of the host wood samples.
- Age-dependent properties are the size-dependent variables of maximal force, fracture surface, and work.
- Analyses via digital image correlation have revealed that most cracks initiate along the mistletoe–host interface with a maximum major strain of about 30%.
- Force–displacement diagrams of intact type samples show successive pre-failure events before they finally fail completely. This pre-failure behaviour is also reflected by high values of deformation at break of about 11% and enables the mistletoe to maintain its structural and functional connection to the host, despite the failure of individual structures.
- The roughness of the fractured surface of intact type samples exhibits a median value of 1.70. The enlarged fracture surface increases the forces necessary to lead to the failure of the connection and contributes to the observed benign fracture behaviour.
- The results of the mechanical study show that the functional and structural integrity of the parasite–host interaction is independent of age (and sex) and thus is guaranteed over the entire lifetime of the mistletoe.

Together with the knowledge from morphological and anatomical studies, we conclude that the risk of sudden failure at the interface between host wood and mistletoe tissue is counteracted by individual sinkers in young mistletoes (pre-failure events) and a lignification gradient that smooths the transition between tissues with differing mechanical properties. Our results increase the understanding of the geometrical and mechanical key characteristics of the interface between the host wood and the fibre-reinforced tissue of the mistletoe. Such knowledge is a prerequisite for the transfer of the concepts deduced from this biological model into the robust interconnections within technical fibre composites, such as carbon fibre-reinforced thermoplastic laminates for structural engineering applications.

## Supplementary data

The following supplementary data are available at *JXB online*.

Dataset S1. Raw data.

Table S1. Detailed statistics on mistletoe sex.

Table S2. Detailed mistletoe statistics.

Table S3. Detailed host wood statistics.

Video S1. DIC video of the nine-year-old mistletoe sample.

erab518_suppl_Supplementary_Table_S1

erab518_suppl_Supplementary_Table_S2

erab518_suppl_Supplementary_Table_S3

erab518_suppl_Supplementary_Dataset_S1

erab518_suppl_Supplementary_Video_S1

## Data Availability

All data supporting the findings of this study are available within the paper and within its supplementary materials published online.
