# Supplementary material for: Biomechanics of the parasite–host interaction of the European mistletoe
Source: J Exp Bot. 2021 Nov 26;73(4):1204–21. doi: 10.1093/jxb/erab518 (PMC8866656; doi:10.1093/jxb/erab518)
Supplement: erab518_suppl_Supplementary_Table_S1 [file erab518_suppl_supplementary_table_s1.pdf]

Mistletoe Sex Statistics
Mistletoe Mechanics
Mylo et al., 2021 - *J. Exp. Bot.*

Variable	Intact type samples: *N* (female) = 26, *N* (male) = 8, *N* (juvenile) = 5; Wilcoxon test (*p* -value)			Sliced type samples: *N* (female) = 25, *N* (male) = 6; Wilcoxon test (*p* -value)
	male vs. female	male vs. juvenile	female vs. juvenile	male vs. female
**Roughness**	0.3267	0.2844	0.1152	0.3913
**Tensile Strength (rough surface)**	0.3901	0.4351	0.9378	0.7888
**Tensile Strength (corresponding surface)**	0.2362	0.8329	0.6203	0.9031
**Fracture Energy (rough surface)**	0.6185	0.2844	0.4168	0.8265
**Fracture Energy (corresponding surface)**	0.6469	0.5237	0.4479	0.5095
**Deformation at Break**	0.3267	0.2844	0.1152	0.3913
**Axial Rigidity**	0.5104	0.01088	0.01584	0.6085
