# Supplementary material for: Biomechanics of the parasite–host interaction of the European mistletoe
Source: J Exp Bot. 2021 Nov 26;73(4):1204–21. doi: 10.1093/jxb/erab518 (PMC8866656; doi:10.1093/jxb/erab518)
Supplement: erab518_suppl_Supplementary_Table_S2 [file erab518_suppl_supplementary_table_s2.pdf]

Detailed Host Statistics
Mistletoe Mechanics
Mylo et al., 2021 - *J. Exp. Bot.*

	**Tesile strength [MPa]**	
	Parallel to grain	Normal to grain
Normal distribution Shapiro-Wilk p-value	0.57	0.919
Variances Levene p-value	0.01	
Differences p-value	Wilcoxon 0.001	
median	19.62	3.43
IQR	9.16	0.65

	**Young's modulus [MPa]**	
	Parallel to grain	Normal to grain
Normal distribution Shapiro-Wilk p-value	0.29	0.952
Variances Levene p-value	0.041	
Differences p-value	Wilcoxon 0.001	
median	660.2	91.3
IQR	319.3	34.3

	**Work/ fracture area [Nm/m^2]**	
	Parallel to grain	Normal to grain
Normal distribution Shapiro-Wilk p-value	0.486	0.062
Variances Levene p-value	0.002	
Differences p-value	Wilcoxon 0.001	
median	7.39	1.50
IQR	5.7	0.44

	**Deformation at break [ ]**	
	Parallel to grain	Normal to grain
Normal distribution Shapiro-Wilk p-value	0.079	0.341
Variances Levene p-value	0.008	
Differences p-value	Wilcoxon 0.002	
median	0.044	0.106
IQR	0.003	0.06

	**Force max [N]**	
	Parallel to grain	Normal to grain
Normal distribution Shapiro-Wilk p-value	0.45	<0.001
Variances Levene p-value	0.02	
Differences p-value	Wilcoxon 0.001	
median	1291.1	124.4
IQR	307.8	11.8

	**Fracture area [mm^2]**	
	Parallel to grain	Normal to grain
Normal distribution Shapiro-Wilk p-value	0.503	0.003
Variances Levene p-value	0.071	
Differences p-value	Wilcoxon 0.112	
median	81.7	37.8
IQR	31.2	11

	**Work [Nm]**	
	Parallel to grain	Normal to grain
Normal distribution Shapiro-Wilk p-value	0.484	<0.001
Variances Levene p-value	0.009	
Differences p-value	Wilcoxon 0.001	
median	0.462	0.05
IQR	0.374	0.009
