# Supplementary material for: Biomechanics of the parasite–host interaction of the European mistletoe
Source: J Exp Bot. 2021 Nov 26;73(4):1204–21. doi: 10.1093/jxb/erab518 (PMC8866656; doi:10.1093/jxb/erab518)
Supplement: erab518_suppl_Supplementary_Table_S3 [file erab518_suppl_supplementary_table_s3.pdf]

Detailed Mistletoe Statistics
Mistletoe Mechanics
Mylo et al., 2021- *J. Exp. Bot.*

Mechanical properties	Statistical dimensions	Intacts		Sections	
		Interface<br>N = 20	Clamps<br>N = 19	Interface<br>N = 13	Clamps<br>N = 18
**Force max [N]**	median	108.8	79.8	241.3	318.7
	IQR	93.8	105.3	103.6	342.8
	normal distribution<br>p-value	<0.001	0.015	0.011	0.111
	correlation with age<br>p-value<br>R^2	Spearman<br>0.12<br>0.379	Spearman<br>0.208<br>0.303	Spearman<br>0.715<br>0.112	Pearson<br>0.033<br>0.504
	variance<br>homogeneity<br>p-value	-		-	
	hypothesis test<br>p-value	-		-	

Mechanical properties	Statistical dimensions	Intacts		Sections	
		Interface<br>N = 20	Clamps<br>N = 19	Interface<br>N = 13	Clamps<br>N = 18
**Fracture area [mm^2]**	median	201.6	158.2	235.6	266.5
	IQR	166.4	134.4	137.9	237.0
	normal distribution<br>p-value	<0.001	0.409	0.190	0.227
	correlation with age<br>p-value<br>R^2	Spearman<br>0.13<br>0.35	Pearson<br>0.01<br>0.578	Spearman<br>0.878<br>0.611	Pearson<br>0.185<br>0.327
	variance<br>homogeneity<br>p-value	-		-	
	hypothesis test<br>p-value	-		-	

Mechanical properties	Statistical dimensions	Intacts		Sections	
**Tensile strength rough surface [MPa]**	median	0.7<br>0.66	0.656	0.89<br>1.143	1.332
	IQR	0.381<br>0.474	0.619	0.433<br>0.781	0.79
	normal distribution<br>p-value	0.014<br><0.001	0.002	0.345<br>0.566	0.126
	correlation with age<br>p-value<br>R^2	Spearman<br>0.497<br>-0.161	Spearman<br>0.801<br>-0.062	Pearson<br>0.166<br>-0.408	Pearson<br>0.376<br>0.222
		Spearman<br>0.444<br>-0.126		Pearson<br>0.537<br>-0.115	
	variance<br>homogeneity<br>p-value	-		-	
	hypothesis test<br>p-value	Wilcoxon<br>0.627		t-test<br>0.221	
		Wilcoxon<br>0.001			

Mechanical properties	Statistical dimensions	Intacts		Sections	
**Tensile strength corresponding area [MPa]**	median	1.201	1.346	1.607	1.691
		1.24		1.61	
	IQR	0.678	2.25	0.702	0.59
		1.651		0.63	
	normal distribution<br>p-value	0.105	<0.001	0.007	0.4
		<0.001		0.05	
	correlation with age<br>p-value<br>R^2	Pearson<br>0.561<br>-0.138	Spearman<br>0.303<br>-0.25	Spearman<br>0.21<br>0.373	Pearson<br>0.57<br>0.144
		Spearman<br>0.184<br>-0.217		Pearson<br>0.45<br>-0.141	
	variance<br>homogeneity<br>p-value	-	-	-	-
	hypothesis test<br>p-value	Wilcoxon<br>0.945		Wilcoxon<br>0.86	
		Wilcoxon<br>0.039			

Mechanical properties	Statistical dimensions	Intacts N = 39<br>Interface N = 20	Intacts N = 39<br>Clamps N = 19	Sections N = 31<br>Interface N = 13	Sections N = 31<br>Clamps N = 18
**Fracture energy rough area [Nmm/mm^2]**	median	0.916	0.772	0.688	0.891
		0.813		0.786	
	IQR	0.968	0.109	0.325	0.338
		0.944		0.399	
	normal distribution p-value	0.118	0.019	0.19	0.447
		0.008		0.22	
		<0.001			
	correlation with age p-value R^2	Pearson	Spearman	Pearson	Pearson
		0.221	0.778	0.878	0.912
		0.292	0.069	-0.047	0.028
		Spearman		Pearson	
		0.452		0.862	
		0.124		-0.033	
		Spearman			
		0.851			
		0.028			
	variance homogeneity p-value	-		0.942	
	hypothesis test p-value	Wilcoxon		t-Test	
		0.366		0.19	
		Wilcoxon			
		0.557			

Mechanical properties	Statistical dimensions	Intacts N = 39<br>Interface N = 20	Intacts N = 39<br>Clamps N = 19	Sections N = 31<br>Interface N = 13	Sections N = 31<br>Clamps N = 18
**Fracture energy corresponding area [J/m2]**	median	1.58	1.73	1.12	1.06
		1.62		1.11	
	IQR	0.96	1.52	0.51	0.59
		1.25		0.54	
	p-value	0.092	0.148	0.014	<0.001
		0.097		<0.001	
	correlation with age p-value R^2	Pearson	Pearson	Spearman	Spearman
		0.327	0.758	0.792	0.843
		0.231	0.075	0.081	0.05
		Pearson		Spearman	
		0.418		0.953	
		0.133		0.011	
	variance homogeneity p-value	0.038		-	
	hypothesis test p-value	Wilcoxon		Wilcoxon	
		0.923		0.679	
		Wilcoxon			
		0.012			

Mechanical properties	Statistical dimensions	Intacts N = 39		Sections N = 31	
		Interface N = 20	Clamps N = 19	Interface N = 13	Clamps N = 18
**Axial rigidity [N]**	median	3904.0	3804.6	11047.0	12676.0
		3864.3		11205.8	
	IQR	5134.6	3539.6	5244.8	9992.3
		4464.0		8299.4	
	normal distribution p-value	0.115	0.02	0.169	0.759
		0.017		0.354	
	correlation with age p-value R^2	Pearson 0.517 0.154	Spearman 0.228 0.290	Pearson 0.653 0.138	Pearson 0.072 0.434
		Spearman 0.211 0.205		Pearson 0.13 0.278	
	variance homogeneity p-value	-		0.148	
	hypothesis test p-value	Wilcoxon 0.989		t-test 0.566	
		Wilcoxon <0.001			

Mechanical properties	Statistical dimensions	Intacts N = 39		Sections N = 31	
		Interface N = 20	Clamps N = 19	Interface N = 13	Clamps N = 18
**Stiffness [N]**	median	183.5	191.4	436.8	291.6
	IQR	160.2	176.5	197.0	291.6
	normal distribution p-value	0.024	0.023	0.383	0.471
	correlation with age p-value R^2	Spearman 0.247 0.271	Spearman 0.095 0.394	Pearson 0.987 0.005	Pearson 0.035 0.499
	variance homogeneity p-value	-	-	-	-
	hypothesis test p-value	-	-	-	-

Mechanical properties	Statistical dimensions	Intacts Interface	Intacts Clamps	Sections Interface	Sections Clamps
**Work [Nm]**	median	0.16	0.09	0.17	0.20
	IQR	0.13	0.10	0.05	0.26
	normal distribution p-value	0.075	<0.001	0.2	0.447
	correlation with age p-value R^2	Spearman 0.289 0.464	Spearman 0.039 0.325	Spearman 0.878 0.23	Pearson 0.912 0.265
	variance homogeneity p-value	-		-	
	hypothesis test p-value	-		-	

Mechanical properties	Statistical dimensions	Intacts Interface	Intacts Clamps	Sections Interface	Sections Clamps
**Deformation at break []**	median	0.117	0.074	0.043	0.061
	IQR	0.056	0.077	0.022	0.021
	normal distribution p-value	0.003	0.0004	0.509	0.004
	correlation with age p-value R^2	Spearman 0.218 0.288	Spearman 0.15 0.343	Pearson 0.074 0.512	Spearman 0.627 0.123
	variance homogeneity p-value	-		-	
	hypothesis test p-value	Wilcoxon 0.127		Wilcoxon 0.01	
