# Supplementary material for: Biomechanics of the parasite–host interaction of the European mistletoe
Source: J Exp Bot. 2021 Nov 26;73(4):1204–21. doi: 10.1093/jxb/erab518 (PMC8866656; doi:10.1093/jxb/erab518)
Supplement: erab518_suppl_Supplementary_Dataset_S1 [file erab518_suppl_supplementary_dataset_s1.pdf]

Raw Data
Mistletoe Mechanics
Mylo et al., 2021- *J. Exp. Bot.*

Age [years]	Sex	Sample	Failure	Max force [N]	Tensile strength (rough surface) [MPa]	Tensile strength (corresponding surface) [MPa]	Deformation at break [%]	Fracture work [Nmm]	Fracture energy (rough surface) [J/m^2]	Fracture energy (corresponding surface) [J/m^2]	Rough fracture area [mm^2]	Corresponding fracture area [mm^2]	Roughness [ ]	Axial rigidity [kN]
8	female	intact	interface	94.30	0.53	0.63	16.72	49.45	1.15	1.37	177.8	149.6	1.19	1.35
8	female	intact	clamp	7.78	0.04	0.07	5.32	52.35	0.06	0.11	210.7	112.2	1.88	1.62
11	female	intact	clamp	254.45	1.61	2.36	16.19	43.58	2.80	4.11	158.2	107.7	1.47	3.20
7	female	intact	interface	128.42	0.51	1.24	8.89	56.71	0.64	1.54	250.3	103.6	2.42	4.53
8	female	intact	interface	218.89	1.27	2.01	8.47	101.28	1.75	2.77	172.0	108.9	1.58	8.77
8	female	intact	interface	172.57	0.59	0.95	8.71	37.36	1.46	2.35	293.1	182.6	1.61	8.19
14	female	sliced	clamp	254.19	1.14	1.38	6.26	50.09	0.79	0.95	222.2	183.7	1.21	14.14
7	female	intact	clamp	11.59	0.06	0.09	23.83	203.43	0.17	0.27	199.4	124.5	1.60	0.15
7	male	intact	interface	57.27	0.70	1.74	13.03	47.37	0.78	1.93	81.6	32.9	2.48	2.41
14	female	sliced	clamp	680.19	1.47	2.26	10.17	462.19	0.89	1.36	461.8	301.0	1.53	13.09
12	female	intact	interface	119.43	0.38	0.93	13.37	285.64	0.53	1.28	310.5	128.3	2.42	1.73
19	female	sliced	interface	367.42	1.09	1.61	8.50	317.52	1.12	1.65	336.3	228.3	1.47	8.82
13	female	intact	interface	573.74	0.70	1.05	27.47	886.50	2.50	3.71	815.5	548.3	1.49	7.14
18	female	sliced	clamp	747.65	1.31	1.61	14.37	691.92	0.91	1.11	571.1	464.5	1.23	21.43
5	juvenile	intact	interface	6.91	0.87	1.52	6.26	204.56	0.54	0.93	7.9	4.6	1.74	0.20
8	female	intact	clamp	103.14	2.16	8.82	2.70	11.74	1.21	4.94	47.8	11.7	4.09	5.45
21	male	sliced	interface	663.56	0.84	1.29	6.48	442.71	0.85	1.30	794.0	515.7	1.54	18.89
12	male	sliced	clamp	159.55	0.62	1.15	5.86	159.56	0.35	0.64	258.9	138.7	1.87	9.60
6	male	sliced	clamp	93.56	1.77	2.53	7.46	301.69	1.00	1.42	52.8	37.0	1.43	4.98
11	female	sliced	clamp	99.00	0.44	0.68	7.19	428.42	0.48	0.74	227.4	145.4	1.56	3.68
9	female	sliced	interface	274.63	2.16	3.75	4.93	33.57	1.54	2.67	127.4	73.3	1.74	11.05
9	female	intact	interface	111.24	2.07	3.31	14.10	63.52	2.27	3.63	53.7	33.6	1.60	4.45
8	female	intact	interface	39.47	0.60	1.57	7.54	164.35	0.52	1.36	65.8	25.2	2.61	1.58
10	female	sliced	interface	85.60	0.73	1.87	5.61	2035.23	0.52	1.33	117.1	45.7	2.56	5.63
11	female	sliced	interface	209.65	0.89	1.61	3.00	4.23	0.69	1.25	235.6	130.5	1.81	9.66
8	male	intact	interface	210.50	0.99	1.80	12.38	57.77	0.89	1.62	213.5	117.0	1.82	7.75
8	male	intact	clamp	113.49	0.66	1.40	13.65	122.09	0.81	1.73	172.0	81.0	2.12	3.36
7	female	intact	clamp	159.68	0.87	1.83	12.48	34.35	1.36	2.85	182.8	87.2	2.10	4.23
9	male	intact	interface	80.57	0.42	0.94	10.92	189.91	0.44	0.97	189.8	85.5	2.22	1.98
3	juvenile	intact	clamp	7.25	0.66	2.99	3.64	139.85	0.37	1.71	11.0	2.4	4.56	0.37
7	female	intact	interface	66.10	0.23	0.40	14.68	248.66	0.57	0.98	285.8	166.9	1.71	6.72
10	male	intact	clamp	40.16	0.14	0.21	7.39	83.29	0.52	0.78	287.8	189.2	1.52	3.80
8	female	intact	clamp	254.37	0.88	1.52	44.28	4.13	1.66	2.85	287.8	167.6	1.72	5.13
7	female	intact	interface	124.19	0.48	0.82	7.44	163.12	0.77	1.31	257.5	151.9	1.70	3.94
8	female	intact	clamp	66.57	0.23	0.61	12.87	148.21	0.31	0.83	294.3	108.8	2.70	9.28
9	female	intact	clamp	31.36	0.29	0.46	16.71	477.50	0.31	0.49	106.9	67.5	1.58	0.78
13	female	intact	interface	83.45	0.70	1.08	9.92	198.51	1.85	2.86	119.6	77.1	1.55	2.94
7	male	intact	clamp	297.07	3.40	6.37	10.36	89.73	1.46	2.74	87.4	46.6	1.88	12.44
12	female	sliced	clamp	297.07	1.61	1.81	10.59	32.98	0.69	0.78	185.0	164.2	1.13	12.26
8	female	sliced	clamp	136.66	0.67	0.81	15.92	220.68	0.83	1.01	205.2	169.1	1.21	1.73
6	male	intact	clamp	103.37	0.82	2.24	5.76	127.88	0.77	2.12	126.8	46.1	2.75	4.72
14	female	sliced	clamp	345.22	1.86	2.05	5.89	97.93	0.90	0.99	186.1	168.0	1.11	11.13
6	female	intact	clamp	95.65	1.72	3.12	6.68	134.05	2.41	4.37	55.6	30.7	1.81	1.55

3	juvenile	intact	interface	11.27	0.92	1.84	29.45	21.14	1.74	3.45	12.2	6.1	1.99	0.19
6	juvenile	intact	clamp	15.50	0.12	0.17	7.37	42.63	0.34	0.46	124.7	93.7	1.33	1.89
9	female	intact	clamp	79.77	0.31	0.51	3.39	41.64	0.16	0.27	260.4	155.6	1.67	6.50
14	female	sliced	clamp	580.56	1.53	2.07	6.68	585.16	1.12	1.52	378.8	280.0	1.35	18.79
9	female	intact	clamp	192.50	0.85	1.23	10.90	80.89	2.58	3.74	226.9	156.3	1.45	12.60
8	female	intact	clamp	46.18	0.57	1.35	6.48	134.89	0.99	2.36	81.5	34.3	2.38	4.50
6	male	intact	interface	106.38	1.11	1.27	4.81	332.73	1.40	1.61	96.2	83.7	1.15	8.18
8	female	intact	interface	187.69	0.71	1.37	14.13	202.40	1.26	2.42	263.5	137.3	1.92	5.78
12	female	sliced	clamp	324.27	1.86	2.31	5.08	41.89	1.20	1.49	174.3	140.3	1.24	18.95
12	female	sliced	clamp	739.25	1.37	1.69	4.31	60.11	1.30	1.60	541.3	437.6	1.24	26.27
17	female	sliced	interface	196.01	0.35	1.42	3.59	174.64	0.26	1.06	562.7	137.6	4.09	11.46
7	female	intact	interface	152.76	0.71	1.16	9.74	409.42	0.94	1.54	214.7	131.3	1.64	3.86
8	male	sliced	interface	314.08	1.37	2.15	6.17	376.68	0.99	1.56	228.5	145.8	1.57	10.05
9	female	sliced	interface	241.32	1.16	1.70	3.07	517.66	0.69	1.01	207.3	141.9	1.46	15.32
9	female	sliced	interface	223.90	0.69	1.16	4.31	672.73	0.58	0.98	324.3	192.9	1.68	8.24
8	male	sliced	interface	155.81	0.84	1.49	4.99	89.21	0.61	1.07	185.4	104.7	1.77	6.51
10	female	sliced	clamp	558.27	1.28	1.69	4.75	52.50	0.92	1.21	435.4	329.9	1.32	21.47
8	female	sliced	interface	262.28	1.01	1.55	3.37	107.99	0.73	1.12	260.3	169.2	1.54	14.06
10	female	sliced	clamp	371.43	1.36	2.49	5.31	195.81	1.78	3.26	274.1	149.1	1.84	13.50
7	male	sliced	clamp	544.84	1.05	1.44	5.60	60.71	1.07	1.47	521.0	378.5	1.38	20.89
6	female	sliced	clamp	198.06	0.57	0.83	5.32	162.78	0.52	0.75	348.9	239.7	1.46	6.48
6	juvenile	intact	clamp	31.29	0.50	0.90	1.80	127.88	0.67	1.21	62.9	34.8	1.81	3.79
8	female	intact	interface	21.79	0.09	0.15	15.22	170.29	0.25	0.42	239.7	143.7	1.67	1.06
12	female	sliced	clamp	286.96	0.65	0.91	3.05	166.46	0.70	0.97	440.5	316.1	1.39	9.57
9	female	sliced	interface	299.72	1.51	2.02	2.49	424.24	0.52	0.70	198.4	148.5	1.34	23.79
8	female	sliced	clamp	313.08	1.56	1.80	7.27	209.59	0.77	0.89	201.2	174.1	1.16	8.70
16	female	sliced	interface	196.16	0.55	0.82	4.97	700.98	0.49	0.73	358.6	239.7	1.50	11.21
-	-	host (tangential)	interface	130.26	3.20	3.65	13.06	146.07	1.21	1.39	40.7	35.7	1.14	4.27
-	-	host (tangential)	interface	113.03	3.80	3.98	11.82	226.96	1.76	1.84	29.8	28.4	1.05	2.92
-	-	host (tangential)	interface	118.48	3.62	3.92	13.52	142.64	1.33	1.44	32.7	30.2	1.08	4.68
-	-	host (tangential)	interface	129.73	3.43	3.78	14.37	189.47	1.50	1.65	37.8	34.3	1.10	4.05
-	-	host (longitudinal)	interface	1291.07	20.63	40.10	4.38	112.36	7.39	14.35	62.6	32.2	1.94	62.61
-	-	host (longitudinal)	interface	937.16	11.47	41.47	4.03	400.45	3.50	12.64	81.7	22.6	3.62	60.00
-	-	host (tangential)	interface	119.83	3.15	3.56	10.65	190.22	2.66	3.01	38.1	33.7	1.13	2.47
-	-	host (tangential)	interface	67.32	2.39	2.84	6.67	486.72	1.33	1.58	28.2	23.7	1.19	2.18
-	-	host (longitudinal)	interface	1019.69	37.18	58.94	3.87	557.68	11.58	18.35	27.4	17.3	1.59	55.90
-	-	host (tangential)	interface	143.21	4.82	5.21	7.42	180.43	1.69	1.82	29.7	27.5	1.08	5.59
-	-	host (longitudinal)	interface	1840.91	19.62	61.16	5.79	306.27	9.45	29.45	93.8	30.1	3.12	58.55
-	-	host (longitudinal)	interface	1327.51	7.19	62.92	4.36	103.73	3.75	32.79	184.5	21.1	8.74	48.18
-	-	host (tangential)	interface	300.77	3.89	4.12	7.04	154.46	2.63	2.79	77.3	73.0	1.06	9.35
-	-	host (tangential)	interface	124.38	2.76	3.08	4.80	174.20	1.05	1.17	45.1	40.4	1.12	6.65
